# Radiotherapy for extensive-stage small-cell lung cancer in the immunotherapy era

**DOI:** 10.3389/fimmu.2023.1132482

**Published:** 2023-08-28

**Authors:** Huanhuan Li, Yangzhi Zhao, Tiangang Ma, Hao Shao, Tiejun Wang, Shunzi Jin, Zhongshan Liu

**Affiliations:** ^1^ Department of Radiation Oncology, The Second Affiliated Hospital of Jilin University, Changchun, China; ^2^ Department of Hematology, The First Hospital of Jilin University, Changchun, China; ^3^ Department of Respiratory and Critical Care Medicine, The Second Affiliated Hospital of Jilin University, Changchun, China; ^4^ NHC Key Laboratory of Radiobiology, Jilin University, Changchun, China

**Keywords:** extensive-stage small-cell lung cancer, radiotherapy, immunotherapy, consolidative thoracic radiotherapy, prophylactic cranial irradiation

## Abstract

Currently, chemoimmunotherapy is the first-line treatment for extensive-stage small-cell lung cancer (ES-SCLC). However, only 0.8%–2.5% of the patients presented complete response after chemoimmunotherapy. Considering that ES-SCLC is highly sensitive to radiotherapy, the addition of radiotherapy after first-line treatment for ES-SCLC could further improve local control, which may be beneficial for patients’ survival. Prior studies have shown that consolidative thoracic radiotherapy (cTRT) can decrease disease progression and improve overall survival in patients with ES-SCLC who respond well to chemotherapy. However, the efficacy and safety of cTRT in the immunotherapy era remain unclear owing to a lack of prospective studies. Prophylactic cranial irradiation (PCI) has been shown to decrease brain metastasis (BM) and prolong survival in patients with limited-stage SCLC in previous reports. However, according to current guidelines, PCI is not commonly recommended for ES-SCLC. Immunotherapy has the potential to reduce the incidence of BM. Whether PCI can be replaced with regular magnetic resonance imaging surveillance for ES-SCLC in the era of immunotherapy remains controversial. Whole brain radiation therapy (WBRT) is the standard treatment for BM in SCLC patients. Stereotactic radiosurgery (SRS) has shown promise in the treatment of limited BM. Considering the potential of immunotherapy to decrease BM, it is controversial whether SRS can replace WBRT for limited BM in the immunotherapy era. Additionally, with the addition of immunotherapy, the role of palliative radiotherapy may be weakened in patients with asymptomatic metastatic lesions. However, it is still indispensable and urgent for patients with obvious symptoms of metastatic disease, such as spinal cord compression, superior vena cava syndrome, lobar obstruction, and weight-bearing metastases, which may critically damage the quality of life and prognosis. To improve the outcome of ES-SCLC, we discuss the feasibility of radiotherapy, including cTRT, PCI, WBRT/SRS, and palliative radiotherapy with immunotherapy based on existing evidence, which may offer specific prospects for further randomized trials and clinical applications.

## 1 Introduction

Small-cell lung cancer (SCLC) accounts for 13%–15% of all lung cancers and is a particularly malignant neuroendocrine carcinoma characterized by rapid growth and trend toward early widespread diffusion (1). Extensive-stage SCLC (ES-SCLC) accounts for 65% of all newly diagnosed cases of SCLC (2). Over the last decade, platinum–etoposide chemotherapy has become the standard chemotherapy. Nevertheless, 75% of the patients have residual lesions in the thorax that commonly recur within the first year (3). Previous prospective trials have suggested that consolidative thoracic radiotherapy (cTRT) improves local control (LC) and overall survival (OS) in patients who respond well to chemotherapy (4–7). Currently, chemoimmunotherapy has become the first-line therapy for ES-SCLC, but only 0.8%–2.5% of the patients presented complete response (CR) (8). These residual lesions lead to disease progression and a poor prognosis. As a local treatment method, cTRT can further improve LC in selected patients and has promising prospects in the era of immunotherapy. Two essential prospective studies (8, 9) that used immunotherapy as first-line treatment for ES-SCLC did not explore the value of cTRT after chemoimmunotherapy. The efficacy and safety of cTRT combined with immunotherapy are controversial because of the lack of randomized trials. However, the benefits of the cTRT remain unclear. The optimal dose of cTRT and the sequence of maintenance immunotherapy and cTRT remain controversial.

In addition, SCLC has a high tendency for brain metastasis (BM). Prior studies have demonstrated that 50% of the patients with SCLC are at risk of evolving to BM after systemic therapy (10). Moreover, chemotherapeutic agents have little effect on preventing BM because of the presence of the blood-brain barrier. The prophylactic cranial irradiation (PCI) could decrease the BM rate and improve OS for patients with limited or extensive SCLC in some investigations (11–13). Nonetheless, the value of PCI for the treatment of ES-SCLC is controversial because of the conflicting conclusions from two prospective studies (11, 14). Furthermore, PCI can decrease the cognitive function of the central nervous system (CNS). Immunotherapy has the potential to reduce BM incidence. Considering the benefits and complications of PCI, it is unclear whether it can be replaced by regular brain magnetic resonance imaging (MRI) surveillance in the era of immunotherapy.

Whole-brain radiation therapy (WBRT) is the standard treatment strategy for BM in SCLC. However, damage to cognitive function and quality of life (QoL) increases with WBRT. Stereotactic radiosurgery (SRS) has shown excellent outcomes in patients with limited BM (15–19). Considering its potential to decrease BM during immunotherapy, WBRT may be replaced by SRS to decrease neurotoxicity in patients with ES-SCLC and limited BM.

Palliative radiotherapy is essential to relieve symptoms and improve the QoL in patients with advanced ES-SCLC. A higher dose per fraction and a shorter total treatment time were appropriate for these patients. Additionally, with the addition of immunotherapy, the role of palliative radiotherapy may be weakened in patients with asymptomatic metastatic lesions. However, it is still indispensable and urgent for patients with obvious symptoms of metastatic disease, such as spinal cord compression, superior vena cava syndrome, lobar obstruction, and weight-bearing metastases, which may critically damage their quality of life and prognosis. However, specific information regarding immunotherapy is still lacking.

However, the efficacy of the existing therapies for ES-SCLC remains limited. As a local treatment strategy, radiotherapy has promising prospects in improving LC and survival. However, evidence regarding the efficacy of radiotherapy for ES-SCLC, especially in the era of immunotherapy, is limited. In this review, we first discuss the efficacy, safety, indications, and dose of cTRT in the immunotherapy era based on existing studies. Second, we explored the benefits and adverse effects of PCI. Considering the potential of immunotherapy to reduce BM, further prospective studies are required to validate whether PCI can be replaced by regular brain MRI surveillance in the era of immunotherapy. Third, we summarized the role of SRS in limited BM in patients with ES-SCLC. SRS may replace WBRT in ES-SCLC patients with limited BM if immunotherapy can decrease BM. Fourth, we discuss the value of palliative therapy for the treatment of advanced ES-SCLC, and explore optimal dose fractionation.

## 2 The consolidative thoracic radiotherapy

### 2.1 The role of consolidative thoracic radiotherapy after chemotherapy alone

Platinum-based chemotherapy has been considered the normal first-line therapy for ES-SCLC in recent decades and has led to a median survival of approximately 10 months (20). Nevertheless, 70%–90% of cases remain residual diseases in the thorax after chemotherapy, and the progression of the resistance commonly occurs in the first year after initial therapy (11). Thus, the local treatment of residual lesions plays an essential role in ES-SCLC. cTRT is considered feasible because of its high sensitivity to irradiation in patients with SCLC. A meta-analysis demonstrated the benefits of OS and progression-free survival (PFS) in patients with ES-SCLC who received cTRT (4). Similarly, a retrospective study indicated that a cTRT dose > 50 Gy was associated with better OS and PFS in patients with ES-SCLC (5). Several prospective studies have demonstrated the significance of cTRT in ES-SCLC (**Table 1**). Jeremic et al. conducted a controlled clinical trial with a dose of 54 Gy/36 fractions twice daily for thoracic radiotherapy with concomitant chemotherapy and further chemotherapy for patients with CR at distant sites. Partial response (PR) at a local site was observed after at least three cycles of chemotherapy in patients with ES-SCLC. The median survival in the cTRT and control groups were 17 and 11 months, and 5-year survival was 9.1% and 3.7% (p=0.041), respectively (6). Patients in the cTRT group showed significantly improved survival rates; however, this trial did not receive much attention. Given the critically enrolled condition, uncommon dose and fraction of cTRT, and the small and single-institution trial in the ‘90s, the widespread generalizability of the investigation remains controversial.

**Table 1:** The prospective trails of thoracic radiotherapy in ES-SCLC.

Study	Time	Number of patients	Patient selection	Dose-fractionation of RT	Median Survival (months)	Local control	PFS	OS
Jeremic et al. (6)	1988 to 1993	109	All patients with ES-SCLC with CR at distant site and PR at local site at least	54 Gy/36 fractions BID chT	17 11	5-year LC: 20.0% 8.1% (*p*=0.006)	1-year PFS: 56.0% 41.0% (*p*=0.045)	5-year 9.1% 3.7% (p=0.041)
RTOG 0937 (21)	2010 to 2016	97	All patients with ES-SCLC with 1-4 metastatic disease and response to chT	30-45 Gy chT	13.8 15.8	NA	Median PFS: 4.9 months 2.9 months (*p*=0.01)	1-year 50.8% 60.1%
CREST trail (7)	2009 to 2012	495	All patients with ES-SCLC with response to chT	30 Gy/10 fractions chT	11.8 7.5	Crude LC: 56.3% 20.2% (p=0.0001)	6-month PFS: 42.0% 7.0% (p=0.001)	2-year 13.0% 3.0%

ES-SCLC, extensive-stage small cell lung cancer; RT, radiation therapy; PFS, progression-free survival; OS, overall survival; CR, complete response; PR, partial response; BID, twice daily; chT, chemotherapy; LC, local control; NA, not available.

A phase II randomized controlled study, RTOG-0937, explored the role of radiotherapy at a dose of 30–45 Gy in intrathoracic lesions and extracranial disease in ES-SCLC. This study enrolled 146 patients with oligometastatic lesions who underwent PCI. However, the study was discontinued in the interim analysis due to invalid OS in both groups. Although the 1-year OS was not significantly different between the two groups (p=0.21), the consolidative radiotherapy group showed a significantly prolonged time to progression (p=0.01). Moreover, the progression at sites of presenting lesions and local regions in consolidated radiotherapy arm compared with the control arm was 41.9% vs. 78.1% and 25.8% vs. 62.5%, respectively. The incidence rate of grade ≥3 toxicity induced by consolidative radiotherapy was higher in the consolidative radiotherapy group than the control group (25% vs. 9.5%) (21). The investigation showed that radiotherapy for intrathoracic disease and extracranial metastases could significantly extend the progression time and change the failure pattern, but could not improve the 1-year OS. The consolidative radiotherapy group had more patients of advanced age with a higher tumor burden, lower CR, and worse performance status (PS), which might lead to a lack of survival benefits.

Furthermore, a randomized CREST trial validated the efficacy of cTRT. In this study, 495 ES-SCLC patients who responded to chemotherapy were included. In the cTRT group, patients received 30 Gy/10 fractions of sequential thoracic irradiation, with PCI allowed in all patients underwent PCI. The 1-year OS, 2-year OS and 6-months PFS in the cTRT arm compared with the control arm were 33% vs. 28% (p=0.066), 13% vs. 3% (p=0.004), and 7% vs. 24% (p=0.001), respectively. Additionally, 43.7% of the patients in the cTRT group exhibited intrathoracic progression compared to 79.8% of the patients in the control group (p<0.0001). Although the 1-year OS was not significantly improved, a significant improvement in the 2-year OS and delayed intrathoracic progression was observed in the cTRT group, suggesting that better LC resulted in improved survival, which is similar to non-small cell lung cancer (NSCLC) (22). The incidence of grade 3–4 toxicities was not significantly different between the two groups. Additional analysis of the CREST trial showed that patients with residual lesions in the thorax after chemotherapy benefited significantly from cTRT (23).

As mentioned above, we consider that the better OS in Jeremic’s study compared to the CREST and RTOG-0937 trials mainly originates from the following reasons: First, the inclusion criterion of the cTRT group in the study by Jeremic et al. was CR at a distant site and PR at a local site, and these selected patients presented better OS than patients with a worse response to chemotherapy, which increased the benefit of OS compared to other studies. An additional analysis of the CREST trial showed that cTRT was beneficial in patients with residual lesions in the thorax after chemotherapy, but not in patients with CR to chemotherapy in the thorax (23). Therefore, patients who respond to chemotherapy, but have residual thoracic lesions, may benefit from cTRT. Second, in Jeremic et al. ‘s study, patients in the cTRT group tended to have less metastatic disease than those in the RTOG-0937 group (the CREST trial did not show relevant data on metastatic disease), which was associated with better OS. The RTOG-0937 trial reported that the survival rate of patients with oligometastatic disease was similar to that of patients with limited-stage SCLC (21). Moreover, a second analysis of the CREST trial revealed that patients with ≤2 metastatic disease had better OS and PFS than those with ≥3 metastases, regardless of whether cTRT was administered, and that patients with ≤2 metastatic diseases receiving cTRT had significantly improved PFS (p = 0.003) (23). Furthermore, a retrospective study indicated that patients with oligometastatic disease without brain or liver metastases could benefit from cTRT for ES-SCLC (24). Patients with residual thoracic disease after chemotherapy and oligometastases (less than two metastatic sites), particularly those without brain/liver metastases at initial diagnosis, might benefit from cTRT. Third, although the fraction schedule of cTRT in Jeremic et al. ‘s study was uncommon, the total dose was higher than that in other randomized trials, which may be associated with better survival. Furthermore, in the CREST trial, patients received a dose of 30 Gy/10 fractions in the cTRT group, and 43.7% of the patients recurred in the intrathorax after cTRT (7), which implies that higher dose of cTRT might be more beneficial. Likewise, several studies have also proposed that a higher dose of thoracic irradiation significantly improves survival (5, 6, 25). Han et al. suggested that a hyperfractionated scheme of 45 Gy/30 fractions during six cycles of chemotherapy was possible (26). Xu et al. suggested that a time-adjusted BED > 50 Gy is related to better survival benefits and tumor control (27). However, complications significantly increased with higher doses. A dose schedule of 30 Gy/10 fractions resulted in lower morbidity and moderate treatment efficacy. The American Society for Therapeutic Radiation Oncology (ASTRO) and American Society of Clinical Oncology (ASCO) clinical practice guidelines recommend radiation doses of 30 Gy/10 fractions to the thorax, while a higher dose (45–54 Gy) is considered to prolong survival (28, 29). The American Radium Society(ARS) recommends a dose of 30–54 Gy for the thorax (30). Therefore, owing to the limited strength of existing evidence, a dose of 30 Gy/10 fractions to the thorax is commonly recommended with acceptable toxicity, whereas a higher dose (45–54 Gy) may prolong survival, particularly in patients with a good PS.

In conclusion, we recommend that patients with residual thoracic disease after chemotherapy and oligometastases (less than two metastatic sites), particularly those without brain/liver metastases at initial diagnosis, should receive cTRT. A dose of 30 Gy/10 fractions is conditionally recommended, and a higher dose (45–54 Gy) in selected patients, such as those with a good PS, is considered to improve survival. Further prospective studies should focus on the optimal dose of cTRT with better survival and tolerable toxicity and whether patients with lower tumor burden could benefit from cTRT better.

### 2.2 The consolidative thoracic radiotherapy in the immunotherapy era

SCLC is characterized by a tobacco-related high mutational burden, which may benefit from immune checkpoint inhibitors (ICIs). In a phase III randomized clinical controlled trial, IMPower 133, atezolizumab combined with carboplatin/cisplatin and etoposide was approved as the first-line treatment standard owing to significant improvements in OS and PFS (8). Likewise, in the CASPIAN trial, durvalumab with platinum-etoposide significantly improved OS compared with platinum-etoposide alone (13.0 months vs. 10.3 months; p=0.0047). Grade ≥3 toxicities in the durvalumab with platinum-etoposide group versus the platinum-etoposide only group were 62% and 62%, respectively, and deaths due to adverse effects were 5% and 6%, respectively (9). The approval of atezolizumab and durvalumab has rendered chemoimmunotherapy a new first-line treatment for ES-SCLC. Similarly, Adebrelimab, a novel programmed cell death ligand 1 inhibitor (PD-L1), with platinum-etoposide chemotherapy significantly improved OS compared to placebo plus chemotherapy (median, 15.3 vs. 12.8 months; HR = 0.72; 95% CI: 0.58–0.9; p=0.0017) with tolerable adverse effects in a phase 3, multicenter, double-blind randomized clinical trial (31). This study has vital implications for the clinical practice of SCLC treatment in China because it included the largest sample size of Chinese patients with ES-SCLC yet. However, this trail enrolled a low proportion of patients aged≥65 years (33%) and of those with BM (2%), which was lower than IMpower33 (8.5%) and CASPIAN (10%). This may be the reason for the longer survival of patients in both experimental and control groups. Meanwhile, a programmed cell death 1 inhibitor (PD-1), Serplulimab, combined with carboplatin/etoposide, prolonged median survival by 4.5 months and decreased the risk of death by 37% with acceptable toxicities, compared to placebo with carboplatin/etoposide (15.4 vs. 10.9 months; HR = 0.63; 95% CI: 0.49–0.82; p<0.001) in the phase 3, international, double-blind randomized clinical trial (32). Moreover, the 2-year OS rate in the serplulimab was 43.1%, which was 5 times higher than that in the control group. Additionally, all secondary outcomes, including the PFS, objective response rate, and duration of response, showed statistically significant improvements not achieved with Impower133 or CASPIAN. The Asian and non-Asian populations did not differ in the subgroup analysis (p=0.58). The results of serplulimab treatment combined with chemotherapy for ES-SCLC are promising. However, the efficacy of chemoimmunotherapy for ES-SCLC is modest, and only 0.8%–2.5% of the patients show CR after first-line chemoimmunotherapy. Generally speaking, lung and regional lymph nodes are the most common sites of recurrence after first-line chemoimmunotherapy, and these patients have a dismal prognosis (33). The residual lesions frequently progress quickly, with a median PFS of 5–6 months even with consolidative immunotherapy, emphasizing the need to improve LC. Combination therapy is a prospective approach for the treatment of ES-SCLS. Radiotherapy can augment tumor cell immunogenicity, regulate signal transduction, and change the inflammatory tumor microenvironment to improve tumor control (34, 35). According to the biological rationale, radiation could promote tumor-specific antigen exposure, which would activate the adaptive immune response, and this response could be amplified further with the addition of immunotherapy. Meanwhile, some patients with ES-SCLC may develop immunotherapy resistance during treatment, and radiotherapy combined with immunotherapy may reverse immunotherapy resistance (33). Dovedi et al. demonstrated that low-dose radiotherapy combined with anti-PD-1 antibodies enhanced LC and tumor recession in a mouse model of colon cancer. The results of next-generation sequencing of T cell receptors showed an increase in T cell infiltration, not only in the irradiated regions but also in the out-of-field irradiated regions, which is termed the abscopal effect (36). Additionally, the abscopal effect has been observed in xenograft models of breast, colon, and pancreatic cancers (37, 38). Therefore, cTRT combined with immunotherapy may exert a synergistic effect in patients with ES-SCLC. However, the value of cTRT in the immunotherapy era is controversial, as it was not included in the IMPower 133 and CASPIAN trials. But a retrospective study including 41 patients with ES-SCLC, 23 patients received 30Gy/10 fractions cTRT after systemic therapy and 18 patients did not receive cTRT, showed that cTRT significantly improved OS compared with systemic therapy only group (1-year OS 78.6% vs. 39.7%, p=0.019 (39). Furthermore, several single-center retrospective studies have also demonstrated a survival benefit of cTRT after first-line chemoimmunotherapy (40, 41). Given that all existing studies are retrospective, the role of cTRT in ES-SCLC in the immunotherapy era should be interpreted with caution, and we look forward to the prospective study results.

Considering the high incidence of pneumonia associated with immunotherapy and radiotherapy, the safety of combination treatments remains unclear. A real-world study showed that cTRT after first-line chemoimmunotherapy did not increase the incidence of adverse events compared with systemic therapy alone (41). A retrospective study involving 36 patients with ES-SCLC who received first-line chemoimmunotherapy followed by cTRT at 60Gy/28 fractions in most patients, showed that 8% of patients developed radiation-related pneumonitis, all of which were grade 1-2, and four patients discontinued immunotherapy after immune-related pneumonitis but completed cTRT (40). Thus, cTRT has a survival benefit and can be tolerated. Whereas, a real-world study including 211 patients with ES-SCLC, of whom 70 received cTRT after first-line chemoimmunotherapy and 141 received systemic therapy only. The results showed that cTRT significantly improved OS (24.1 months vs. 18.5 months, p=0.016) and PFS (9.5 months vs. 7.2 months, p=0.009) compared with systemic therapy alone. However, the incidence of treatment-related pneumonitis (p=0.018) was significantly increased in cTRT group, most of which were grade 1-2. Since the current studies on the value and toxicity of cTRT in ES-SCLC are single-center retrospective studies, the patients enrolled are heterogeneous, and there is selection bias, which should be interpreted with caution. The PACIFIC randomized clinical trial explored the effectiveness of durvalumab in patients with unresectable stage III NSCLC after concomitant chemoradiotherapy. The durvalumab arm showed significantly improved OS compared with the control arm (p=0.0025). The toxicities of grade 3/4 pneumonia in the durvalumab arm compared with the control arm were 4.4% and 3.8%, respectively (10, 42). Thus, concomitant chemoradiotherapy followed by durvalumab treatment significantly prolonged OS and was associated with a slight increase in complications. Similarly, in a phase I study, thirty-eight patients with ES-SCLC received cTRT immunotherapy after chemotherapy. Only two patients experienced grade 3 toxicities and no grade 4/5 adverse effects (43). Similarly, previous studies have shown that cTRT with immunotherapy is safe and effective for treating NSCLC (44, 45). However, in the PEMBRO-RT study, pneumonia tended to occur in the pembrolizumab with radiotherapy group compared to the pembrolizumab-only group (26% vs. 8%, p=0.06) (46). An analysis of three prospective studies including 26 patients with ES-SCLC who received thoracic radiotherapy (45 Gy/15 daily fractions) combined with chemotherapy and pembrolizumab, 25 patients with limited-stage SCLC who received thoracic radiotherapy (45 Gy/30 twice-daily fractions) with chemotherapy and pembrolizumab, and 27 patients with NSCLC who received thoracic radiotherapy (45 Gy/15 daily fractions) with pembrolizumab showed safety and feasibility of thoracic radiotherapy combined with immunotherapy in the short term. Only three patients experienced pulmonary-specific grade ≥3 toxicity in 53 patients (n=26 SCLC, n=27 NSCLC) who received 45 Gy/15 fractions of thoracic radiotherapy combined with immunotherapy (47).

Preclinical and clinical studies have indicated that cTRT combined with immunotherapy is safe and effective. We believe that patients with residual thoracic disease after first-line therapy and oligometastases (less than two metastatic sites), particularly those without brain/liver metastases at initial diagnosis, might benefit from cTRT in the immunotherapy era. However, evidence is limited, and prospective studies are still lacking. A phase II/III randomized clinical trial, NRG LU007 (NCT04402788), will enroll patients with ES-SCLC without progression after 4–6 cycles of platinum/etoposide/atezolizumab and randomize them to the atezolizumab maintenance group or atezolizumab maintenance combined with radiotherapy (up to five lesions including primary disease in the thorax) group. In addition, the number of disease (1–3 vs. ≥3), PS, and PR status vs. stable disease after first-line treatment were regarded as stratification factors. This trial will validate whether radiotherapy plus immunotherapy can improve treatment efficacy without significantly increasing toxicity after first-line chemotherapy. In addition, a phase II randomized clinical controlled trial (NCT04462276) explored the efficacy and feasibility of cTRT (a dose of 30 Gy/10 fractions) combined with atezolizumab maintenance versus atezolizumab maintenance only after four cycles of platinum/etoposide/atezolizumab therapy for ES-SCLC. Regarding the cTRT dose, as aforementioned, the scheme of 30 Gy/10 fractions was safe and tolerable, particularly considering that immunotherapy can induce pneumonia. When a higher dose is considered, physicians should cautiously weigh the survival benefit and PS of the patients. The optimal dose of cTRT remains unclear because of the lack of prospective studies in the immunotherapy era. In addition, the sequence between cTRT and immunotherapy maintenance remains unclear. A retrospective study indicated no significant benefit of early thoracic radiotherapy (≤ 3 cycles of chemotherapy) compared to late thoracic radiotherapy (> 3 cycles of chemotherapy) (48). Han et al. proposed that early thoracic radiotherapy (within 6 cycles of chemotherapy) could enhance LC. The ASCO guidelines recommend a dose of 30 Gy/fraction of cTRT within 6–8 weeks of chemotherapy and prior to immunotherapy maintenance. However, cTRT was administered during immunotherapy maintenance in the NCT04462276 and NCT04402788 trials. We believe that ES-SCLC is a systematic disease, and chemoimmunotherapy is the primary treatment approach, which differs from NSCLC. cTRT during immunotherapy maintenance could further improve LC in patients with residual thoracic tumors after induction chemoimmunotherapy, and patients could tolerate it well.

In conclusion, a dose of 30 Gy/10 fractions of cTRT during immunotherapy maintenance may be safe and efficient for ES-SCLC patients with lower tumor burden who exhibit regression but show residual thoracic disease after chemoimmunotherapy. Prospective studies NCT04462276 and NCT04402788 will further validate the efficacy and toxicity of cTRT in the immunotherapy era. Further clinical trials should focus on determining the optimal cTRT dose and the sequence of cTRT and immunotherapy maintenance.

## 3 The prophylactic cranial irradiation

### 3.1 The role of prophylactic cranial irradiation

SCLC is an aggressive malignant disease associated with a high risk of BM development. Previous studies have shown that 50% of the patients with SCLC are at risk of evolving to BM after chemotherapy (10, 44). Additionally, antitumor agents are less likely to prevent BM because of the presence of the blood-brain barrier. Therefore, PCI plays an essential role in decreasing BM and improving survival. A meta-analysis of seven clinical trials conducted between 1965 and 1995 enrolled 987 patients who achieved CR after chemotherapy for ES-SCLC or limited-stage SCLC. Compared with the observation arm, PCI reduced 3-year incidence of BM (p<0.001) and increased 5.4% 3-year OS rate (p<0.001) (12). Similarly, another meta-analysis conducted by Meert et al. included 1,547 patients with limited or extensive SCLC in 12 clinical trials. PCI reduced BM incidence in all trials (hazard ratio (HR), 0.48; 95% CI, 0.39–0.60) and improved survival in patients with CR to chemotherapy (HR, 0.82; 95% CI, 0.71–0.96) (13).

The aforementioned studies have validated that PCI can reduce BM and prolong survival. However, considering that brain MRI was not commonly performed in these studies, the NCCN guideline updated PCI recommendation for LS-SCLC from category 1 to category 2A and added adjuvant PCI recommendations for ES-SCLC in 2022 (49) The controversary of PCI for ES-SCLC owing to two clinical trials with conflicting results recently (**Table 2**). The European Organization for Research and Treatment of Cancer (EORTC) reported a stage III randomized clinical trial of patients with ES-SCLC who responded to four to six cycles of chemotherapy. A dose of 20–30 Gy/5–12 PCI fractions was delivered to patients in the PCI group. The incidence of BM and 1-year OS were 14.6% and 40.4% in the PCI group, respectively, and 27.1% and 13.3% in the control group, respectively (11). This study reveals the benefits of PCI in decreasing BM and improving OS in patients with ES-SCLC. However, another randomized clinical trial conducted in Japan included 224 ES-SCLC patients who responded to chemotherapy. Patients in the PCI group received 25 Gy/10 fractions of brain irradiation. Unfortunately, the study was terminated in the interim analysis because of inconsistent results between the two groups. Although patients in the PCI group failed to prolong survival, the incidences of BM at 18 months were 40.1% and 63.8% in the PCI and control groups, respectively (14). The conflicting results of these two studies stem mainly from the following factors. First, brain MRI is necessary in Japan, both before PCI and during follow-up. In the European Organization for Research and Treatment of Cancer (EORTC) study, patients with CNS symptoms underwent brain imaging before PCI and during follow-up. Consequently, only 27% of the patients received brain CT/MRI to evaluate CNS in the EORTC study. It is possible that many patients with BM were randomized to the observation group, which resulted in undertreatment and further supported the benefits of PCI. Second, during follow-up, regular brain MRI is mandatory in Japan’s study, whereas patients who experienced CNS symptoms could receive brain CT/MRI in the EORTC study. Hence, 83% of the patients in Japan’s study experienced salvage radiotherapy for BM compared to 59% of the patients in the EORTC study. Regular brain MRI surveillance and timely salvage radiotherapy do not benefit patients undergoing PCI in Japan. Thus, we conclude that PCI improves the survival of patients without regular brain MRI surveillance for ES-SCLC but does not seem to prolong the survival of patients with regular brain MRI surveillance and timely salvage radiotherapy.

**Table 2:** The key randomized trials of PCI in ES-SCLC.

Study	Time	Number of patients	Arm	Medan Survival (months)	Median PFS (months)	1-year OS (%)	1-year OS (%)	1 year risk of BM (%)
EORTC trial (11)	2007	286	PCI	6.7	3.5	27.0	NA	14.6
			No PCI	5.4	2.8	13.0		40.4
				*p*=0.003	*p*=0.02			*p*<0.0001
Takahashi et al. (14)	2017	224	PCI	11.6	2.3	48.4	15.0	32.9
			No PCI	13.7	2.4	53.6	18.8	59.0
				*p*=0.09	*p*=0.75			

PCI, prophylactic cranial irradiation; ES-SCLC, extensive-stage small cell lung cancer; PFS, progression-free survival; OS, overall survival; BM, brain metastases; NA, not available; EORTC, European Organization for Research and Treatment of Cancer.

In addition, PCI induces neurocognitive toxicity. Prior studies have demonstrated that PCI is associated with >3 times the risk of neurocognitive recession (14), whereas other studies have reported apparent neurotoxicity after PCI (50, 51). With the advancement of modern radiotherapy technology, hippocampal-avoidance whole-brain radiation therapy (HA-WBRT) has been used in clinical practice. The NRG Oncology CC001 trial indicated that compared to WBRT combined with memantine, HA-WBRT combined with memantine significantly decreased cognitive function damage without compromising treatment efficacy in patients with BM (p=0.02) (52). In a phase III clinical study, 150 patients with SCLC were enrolled and randomized into hippocampal avoidance-prophylactic cranial irradiation (HA-PCI) and PCI arms. The HA-PCI arm significantly protected cognitive function and presented no difference in therapeutic effectiveness compared with the PCI arm (p=0.003) (53). However, a phase III randomized trial validated that HA-PCI did not improve cognitive deterioration compared to conventional PCI (p=1.000) (54). These conflicting results render the role of HA-PCI unclear. An ongoing phase II/III randomized clinical study of NRG CC003 for SCLC will validate whether HA-PCI can reduce cognitive function deterioration without compromising treatment efficacy. The NCCN panel believed that HA-PCI using IMRT might be a promising approach for reducing cognitive deterioration (49). Furthermore, the NCCN proposed that PCI should not be recommended for patients with poor PS (3-4) or impaired neurocognitive function owing to neurotoxicity induced by PCI. Additionally, elderly patients (> 60 years) have been related to delayed neurotoxicity (55, 56). Therefore, PCI should be performed with caution in these patients. It should not be performed until the acute toxicities of the initial treatment are resolved.

In conclusion, the role of PCI in ES-SCLC is uncertain considering the lack of high-level evidence. However, routine brain MRI surveillance is necessary to determine whether or not PCI should be performed. HA-PCI using IMRT may be a promising approach for reducing cognitive deterioration in patients with ES-SCLC. PCI is not recommended for patients with a poor PS (3/4) or impaired neurocognitive function. Physicians should be cautious about whether or not PCI should be administered to the elderly patients (> 60 years).

### 3.2 Optimal dose of prophylactic cranial irradiation

A total of 25 Gy/10 fractions is the recommended standard dose. A randomized clinical investigation, EORTC 22003-08004, explored the optimal dose of PCI by enrolling 720 patients with limited-stage SCLC and randomizing them to a standard dose arm of 25 Gy/10 fractions and a higher dose arm of 36 Gy/18–24 fractions. The 2-year BM rate and OS in the standard dose arm vs. and higher dose arms were 29% and 23% (p=0·18) and 42% and 37% (p=0·05), respectively (57). Additionally, the high-dose group exhibited significant toxicity. Therefore, PCI with a total dose of 25 Gy/10 fractions is the optimal regimen for limited-stage SCLC. The Expert Panel on Radiation Oncology in America recommends PCI of 25 Gy/10 fractions or MRI surveillance every 3 months for patients who respond to initial treatment and do not develop BM (30). In addition, some patients with ES-SCLC received 20 Gy/5 fractions of PCI in randomized clinical trials, with impressive results of decreased BM and improved survival (11). The NCCN panel considered that select patients might benefit from a shorter course (49). Future studies should explore this issue further. Therefore, PCI of 25 Gy/10 fractions may be reasonable for patients with ES-SCLC, and for select patients (e.g., those with shorter survival), a shorter course (20Gy/5 fractions et al.) may be considered.

### 3.3 The prophylactic cranial irradiation in the immunotherapy era

Given the unclear survival benefits and CNS toxicity, the value of PCI for ES-SCLC remains controversial, particularly in the era of immunotherapy. As immunotherapeutic drugs can pass through the blood-brain barrier, if immunotherapy decreases BM, PCI may be omitted from future clinical practice. In PACIFIC, a randomized clinical controlled trial for NSCLC, the immunotherapy group reduced the incidence of new BM compared to the control group (6.3% vs. 11.8%) (53). Additionally, one study reported that ipilimumab combined with nivolumab is clinically effective in treating BM (58). Immunotherapy appears to have a positive effect on BM treatment. However, the IMpower-133 study did not provide data on the new BM. Therefore, it remains unclear whether atezolizumab decreases the incidence of new BM. An open phase III clinical trial, the SWOG S1827 study or MAVERICK study (NCT04155034), will explore the value of MRI surveillance versus PCI in patients with limited-stage SCLC or ES-SCLC undergoing systemic therapy. Immunotherapy was used as a stratification factor for ES-SCLC in this study. This study further validated whether regular MRI surveillance could replace PCI for ES-SCLC in the immunotherapy era.

## 4 Whole brain radiation therapy versus stereotactic radiosurgery

More than 18% of the patients with SCLC have BM at first diagnosis (59). The prognosis is dismal, with a median OS of 4–5 months in patients with SCLC and BM (60). A dose of 30 Gy/10 fractions of WBRT is recommended for patients with SCLC who experience BM (15). However, to decrease the decline in cognitive function and QoL induced by WBRT, more studies have focused on SRS. Currently, SRS has become the new standard of treatment for patients with limited BM because prospective studies have revealed that adding WBRT to SRS does not significantly improve OS, with an increase in complications (15–17). It is worth noting that SCLC was excluded from the milestone RTOG randomized trials, which established the SRS as the standard for limited BM (61). However, a meta-analysis including nine retrospective studies that enrolled 1,638 patients with SCLC with limited BM presented a significant benefit of SRS for these selected patients. The 6- and 12-month OS in the SRS arm compared in the WBRT arm were 67% vs. 57% and 39% vs. 29% (p<0.001). Moreover, the 12-month LC rate for SRS was 93% (95% CI], 91 - 94%). In addition, SRS plays a vital role in salvage therapy when BM recurs after WBRT (18). A multicenter retrospective study, including data from 28 centers and a single-arm trial, enrolled 710 patients with SCLC who received first-line SRS without PCI or WBRT. This study recommends SRS as a first-line therapy for selected patients with SCLC and BM, especially for those with single BM (19). This study shows promising prospects for SRS in patients with limited BM.

In addition, T cells can exert positive anti-tumor effects in the brain with immunotherapy for BM in patients with NSCLC or melanoma (58, 62, 63). These trials have validated that immunotherapeutic agents can reduce BM. Furthermore, the immune microenvironment was positive for BM from SCLC, with 75% PD-L1 expression in samples, which indicates that immunotherapy may be effective for patients with SCLC with BM (64). In two clinical trials, durvalumab (HR = 0.79; 95% CI: 0.44-1.41) has shown OS benefit for patients with baseline BM in SCLC, while atezolizumab (HR = 0.96; 95% CI: 0.46-2.01) has not (65, 66). A meta-analysis indicated that patients with ES-SCLC and BM did not benefit from immunotherapy combined with chemotherapy. Nevertheless, the proportion of patients who progressed to BM was similar between the durvalumab and control groups (11.6% vs. 11.5%) in the CASPIAN trial, which did not administer PCI in the durvalumab group. Considering that only 9%-14.2% of the patients with BM were enrolled in the chemoimmunotherapy group in the phase II/III clinical trials and wide CI, the value of immunotherapy for patients with baseline BM remains uncertain. SRS with immunotherapy improved intracranial LC and survival without significantly increasing toxicities in NSCLC, melanoma, or renal cell carcinoma (67–69). However, the neurotoxicity caused by radiotherapy combined with immunotherapy is noteworthy. Although immunotherapy does not often induce neurotoxicity, it is frequently caused by radiotherapy, particularly radiation necrosis, which commonly occurs during and after treatment. Radiation necrosis may be associated with radiotherapy and immunotherapy (70–74). However, a retrospective study suggested that brain radiotherapy combined with immunotherapy did not significantly raise neurotoxicity (74). Therefore, SRS combined with immunotherapy may produce a synergic effect in patients with SCLC with limited BM. However, neurotoxicity caused by combination therapy is of great concern. Early administration of immunomodulatory agents or corticosteroids may decrease toxicity.

The value of SRS for SCLC is controversial owing to the low level of evidence. WBRT remains the standard therapy for BM in patients with SCLC. Further prospective studies are required to validate whether SRS combined with immunotherapy can replace WBRT in patients with limited BM and acceptable adverse effects.

## 5 Palliative radiotherapy

Palliative radiotherapy is used to relieve symptoms and improve QoL. Palliative radiotherapy for ES-SCLC is commonly used for patients with superior vena cava syndrome, lobar obstruction, bone metastasis, and spinal cord compression. For these patients, a higher dose per fraction and shorter total treatment time are vital for rapid alleviation of symptoms. The common dose fractionation scheme included 30 Gy/10 fractions, 20 Gy/5 fractions, and 8 Gy/1 fraction (49). With the addition of immunotherapy, the role of palliative radiotherapy may be weakened in patients with asymptomatic metastatic lesions. However, it is still indispensable and urgent for patients with obvious symptoms of metastatic disease, such as spinal cord compression, superior vena cava syndrome, lobar obstruction, and weight-bearing metastases, which may critically damage their QoL and prognosis. Moreover, conformal techniques such as intensity-modulated radiation therapy are necessary to reduce complications. Making an excellent treatment scheme requires a multidisciplinary team to comprehensively consider the direct and potential benefits for patients. In addition, a specific expectation assessment is required. Finally, treatment plans should consider the care problems in patients with advanced disease. Currently, specific statements regarding palliative radiotherapy in the immunotherapy era are lacking, and more clinical trials should focus on this area.

## 6 Conclusions and perspectives for clinical practice

Previous trials have shown that consolidation radiotherapy can extend OS and delay disease progression after chemotherapy. However, no prospective trials have demonstrated the efficacy or safety of consolidated radiotherapy in the immunotherapy era. We drew the following conclusions from our literature review:

1. A dose of 30 Gy/10 fractions of cTRT is recommended for patients with ES-SCLC with a good response after initial system treatment but who still have residual thoracic disease, particularly without liver/brain metastases. When the patient could tolerate it, the dose was increased to 45–54 Gy.
2. A dose of 25 Gy/10 fractions of PCI could be used to prevent BM in patients with ES-SCLC who are considered to have a longer life span but is not recommended for patients > 60 years of age, with poor PS or impaired neurocognitive function. HA-PCI can also be used to reduce neurotoxicity. In addition, regular brain MRI is necessary.
3. WBRT at a dose of 30 Gy/10 fractions is the standard therapy for BM in patients with SCLC. Furthermore, SRS can be considered for patients with limited BM.
4. Dose fractionation schemes, including 30 Gy/10 fractions, 20 Gy/5 fractions, and 8 Gy/1 fraction of palliative radiotherapy, should be applied individually to alleviate symptoms and improve patient QoL.

Although immunotherapy has changed the therapeutic modality for ES-SCLC, the OS for ES-SCLC patients with ES-SCLC who have undergone chemoimmunotherapy is not ideal. Additionally, PD-L1 expression and the tumor mutation burden failed to predict the efficacy of immunotherapy (65, 75). Thus, effective biomarkers should be explored to identify patients who could benefit from chemoimmunotherapy. Moreover, patients with SCLC had a median age of 71 years at diagnosis in a real-world setting (76), which was older than the patients enrolled in the phase II/III clinical trial (≤ 65 years). The proportion of the elderly patients (≥ 70 years) is increasing (77). Whether or not patients with SCLC aged > 65 years can benefit from immunotherapy is also controversial. Among elderly patients, 60% had a PS of 2, which is challenging for chemoimmunotherapy. The phase II SPACE trial (NCT04221529) and phase III MAURIS trial (NCT04028050) recruited patients with a PS of 2. Future studies are required to demonstrate whether or not these patients could benefit from chemoimmunotherapy. In addition, due to the immunosuppressive microenvironment of SCLC, the efficacy of chemoimmunotherapy is limited, and novel treatment strategies or immunotherapy schemes for ES-SCLC merit further investigation.

## Author contributions

ZL has provided the conception. HL has written the first and revised draft. YZ, TM, HS, TW and SJ have revised the draft and provided substantial input. All authors contributed to the article and approved the submitted version.
